# The Study of Drag Reduction on Ships Inspired by Simplified Shark Skin Imitation

**DOI:** 10.1155/2018/7854321

**Published:** 2018-05-02

**Authors:** M. D. Ibrahim, S. N. A. Amran, Y. S. Yunos, M. R. A. Rahman, M. Z. Mohtar, L. K. Wong, A. Zulkharnain

**Affiliations:** ^1^Department of Mechanical and Manufacturing Engineering, Universiti Malaysia Sarawak, Kota Samarahan, Malaysia; ^2^Faculty of Resource Science and Technology, Universiti Malaysia Sarawak, Kota Samarahan, Malaysia

## Abstract

The skin of a fast swimming shark reveals riblet structures that help reduce the shark's skin friction drag, enhancing its efficiency and speed while moving in the water. Inspired by the structure of the shark skin denticles, our team has carried out a study as an effort in improving the hydrodynamic design of marine vessels through hull design modification which was inspired by this riblet structure of shark skin denticle. Our study covers on macroscaled design modification. This is an attempt to propose an alternative for a better economical and practical modification to obtain a more optimum cruising characteristics for marine vessels. The models used for this study are constructed using computer-aided design (CAD) software, and computational fluid dynamic (CFD) simulations are then carried out to predict the effectiveness of the hydrodynamic effects of the biomimetic shark skins on those models. Interestingly, the numerical calculated results obtained show that the presence of biomimetic shark skin implemented on the vessels give about 3.75% reduction of drag coefficient as well as reducing up to 3.89% in drag force experienced by the vessels. Theoretically, as force drag can be reduced, it can lead to a more efficient vessel with a better cruising speed. This will give better impact to shipping or marine industries around the world. However, it can be suggested that an experimental procedure is best to be conducted to verify the numerical result that has been obtained for further improvement on this research.

## 1. Introduction

Shipping industry has been a thriving industry, since the start of the industrial revolution, and now, it is categorized as one of the large scale economies [1]. Liner ship is facing an unexpected development on the size of the vessels as it has increase endlessly year after year. The escalation in vessel size decreases both fuel and capital cost per cubic capacity required by it [1, 2]. Flow over bodies, either air or fluid, is commonly encountered in practice, and it is normally responsible for numerous physical phenomenon such as hydrodynamic drag especially for immersed transportations like ships [3]. Reducing this resistance may help in improving cruising speed and reduce fuel consumption as well [3, 4]. Scientists today have come up with numerous studies attempting to overcome this the problem, which includes the study of shark skin for drag reduction, self-cleaning super hydrophobic surface originated from the uniqueness of water repellency properties of lotus leaves, and drag reduction through micro bubble injection on ships [5, 6].

Our focus in this study is on hull form design modification through biomimetic riblet structure of shark skin denticle as an attempt of improving the hydrodynamic design of marine vessels. However, in this present paper, we described our approach by focusing on wall shear, velocity profile, and turbulence kinetic energy (TKE) produced by the modified and unmodified hull form through CFD activity. Macroscaled biomimetic riblet shark skin is applied on the frontal and rear vicinity of the container ship with the intention of improving the fluid flow around the ship which will provide better flow separation control especially at the ship hull surface area. Endless studies have been carried out recently as an effort upon improving performance of systems such as ship performance, duct or pump efficiency, and energy saving. However, these studies focus on the nanoscaled modifications, which require more financial resources in order to be implemented due to the complex structure of the shark skin denticles. The studies include the investigation on drag-reduction mechanism which are inspired by nature such as drag reduction through the unique structure of shark skin denticle, hydrophobic surface inspired by lotus leaves, and drag reduction by injections of micro bubbles on ships which sometimes might lead to cavitation problems [5, 6]. Mimicking biological structure such as shark skin denticle as a mechanism to reduce drag resistance is an example of how nature can inspire us to come up with ideas that can provide similar function as them. As explained by most researchers, it is proven that the unique structure of the shark skin denticles gives hydrodynamic effect that can help in reducing drag. For example, various studies have been carried out by many researchers which discuss on drag-reduction mechanism either by using model which was built from data processing via high-accurate scanning based on real biological shark skin or by using simplified version of shark skin through several riblet designs or geometries for various applications including the study in rectangular duct flow and riblets on airfoil design [7, 8]. Our design, however, proposes a much more economical and practical macroscaled modifications to obtain a better ship cruising characteristics with a relatively cheaper solution and gain fuel savings eventually in return.

### 1.1. Shark Skin Denticle and the Principal Effect

**Figure 1:**
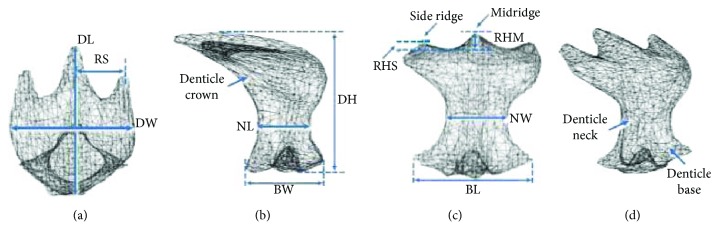
3D reconstructed micro-CT model of a single denticle of mako shark [9].

Shark skin structures are rough and harsh, and they are made up of dermal tooth-like denticle which is called as miniscule placoid scales or denticles. These denticles have pulp and cavity covered with enamel or vitrodentine [9, 10]. Placoid scale varies vastly and has a crown-shaped neck and base. The scales are in series of parallel ridges which majorly exist in faster species sharks such as shortfin mako shark, “*Isurus oxyrinchus*.” These ridges are beneficial in surface roughness by providing a hydrodynamic effect [11].  Figure 1 shows a 3D reconstructed micro-CT model of a mako shark denticle which point at the middle trunk position by Wen et al. in their study on the mimicry of shark's denticle. Based on the study, Figure 1(a) is the top view of the denticle; Figure 1(b) is the lateral view, where DH, denticle height; BW, base width; and NL, neck length; Figure 1(c) is the anterior view, where RHS, height of side ridge; RHM, height of midridge between two dashed line; NW, neck width; and BL, based length of the denticle; Figure 1(d) shows the morphological measurement provided in Figures 1(a)–1(c). In Figure 1(a), DH is the denticle height, BW is the base width, and NL is the neck length; in Figure 1(b), DH is the denticle height, BW is the base width, and NL is the neck length; in Figure 1(c), RHS is the height of the side ridge, RHM is the height of the midridge between two dashed line, NW is the neck width, and BL is the based length of the denticle. The investigation covers the study on the design by fabricating it using 3D printing which imitates the real skin of shortfin mako shark using micro-CT imaging and studies the hydrodynamic effect of the shark skin. The result of this investigation shows that the 3D-printed shark skins give a significant 6.6% increase in speed-saving energy up to 5.9% [9].

This is considered as a natural phenomenon occurred in living animals. Principally, the existence of these denticles helps to maintain the smooth flowing water in laminar pattern resulting in faster speed of fluid close to the surface of a shark body. This helps in reducing the velocity difference between near surface and away fluid flow from surface during locomotion in water. This velocity difference sustains the pattern of the fluid when it travels along the body of the shark before it splits of into turbulence flow. Thus, resulting in a lesser drag force and faster speed during swimming in a much lesser effort [12].

### 1.2. Mechanism of Fluid Drag

Fluid flow over bodies commonly occurs in real life and normally leads to physical phenomenon such as drag force which frequently happened in transportations, lift force in the wings of an airplane, and upward draft during raining or snowing and effects of high winds to dust particles. Pressure drag and friction drag are the two most basic type of fluid drag. Pressure drag can be envisioned by a cross-flow over an object. It requires power or energy needed to move the front object out from the fluid in contact and then return back behind the object. Streamlining an object can help in reducing pressure drag [4, 12]. Friction drag or viscous drag is normally associated between the interactions of fluid with the surface which is parallel to the flow of the fluid. Theoretically, the fluid is in no-slip condition at the surface of the object where velocity is said to be zero and each neighbouring layer which is parallel to that surface will be faster as they get further away from the surface until each layer reaches the similar speed to the free stream velocity. The energy required to transfer the momentum in a velocity gradient for the interaction between surface and surrounding fluid is what defined as friction drag [4].

The relationship between drag and the velocity of the body is commonly described by the given equation: drag force, *F*_D_ = 1/2*ρAC*_d_*V*^2^. Where *C*_d_ is the drag coefficient of the object relative to its shape, structure, orientation, and size. From the given equation of *F*_D_, the force acting on any object is proportional to the square of the fluid's speed, the density of the fluid, projected areas of the fluid, and the drag coefficient, *C*_d_, of the object which encountered by the fluid. Since *F*_D_ is proportional to drag coefficient, thus the decrease in *C*_d_ value will reduce the amount of force that acts toward the body provided that all other parameters are constant. The aim of our study is to reduce drag force exerted on the body by doing modification on the hull form through riblets which mimic the structure of the shark skin denticle. With the help of powerful software like computational fluid dynamic (CFD), ANSYS will calculate the drag coefficient on both models, modified and unmodified hull forms. The amount of force acting on the body will be obtained through drag force equation. In this study, the graph of pressure along modified and unmodified ships is plotted in Section 3. Pressure is proportional to the force acting on the object; hence, high pressure results in high force exertion.

#### 1.2.1. Four Factors Effecting Drag

Basically, there are four factors that affect drag force. These are velocity or speed, the size or length of the object or solid, the density of the fluid, and lastly, the viscosity of the fluid. However, the transition from laminar to turbulent flow also depends on other factors such as surface geometry, surface roughness, the upstream velocity, the type of fluid, and the temperature of the surface. The relationship of these four factors is used in order to determine the type of flow either laminar or turbulence which can be calculated using Reynolds number (Re) [4]. Reynolds number can be expressed as Re = *ρvL*/*μ*. Where *ρ* is the density of fluid (kg/m^3^), *L* is the length of the object (m), *v* is the velocity (m/s), and *μ* is the viscosity of the fluid (kg/m·s).

Generally, drag force, *F*_D_, that acts on an object depended on the following given quantities: velocity (*v*), density of the fluid (*ρ*), viscosity (*μ*), and the size or length of the object (*L*). The relationship of these four factors functions to determine the type of flow either laminar or turbulence which can be calculated using the Reynolds number, Re = *ρvL*/*μ*. However, the transition from laminar to turbulent flow also depended on other factors such as surface geometry, surface roughness, upstream velocity, and type of fluid as well as the surface temperature depending on type of case study [4]. It is important to identify the type of flow by calculating the Reynolds number as it is used to determine the flow type expected in any given condition. It helps in predicting the type of flow patterns either laminar, transition, or turbulent flow, thus acts as a measuring tool for engineers to do modifications or optimization on designs to improve the flow pattern. For fluid flow over a smooth plate, the transition of flow exists in laminar to turbulent which begins at Re = 1 × 10^5^ but still does not turn to a complete turbulent flow when Re reaches Re = 3 × 10^6^ [4]. Thus, the correct selection of flow type in the numerical method through CFD activity is important to obtain a more accurate prediction relative to the real condition.

### 1.3. Design Altering for Optimization

Enhancement of functional properties of any mechanism today is made possible by altering or improving its original design. The act of modifying the design of any geometries will contribute significantly to the performance of the system either dynamically or statically. Ibrahim et al. describe on how geometrical optimization on the design of conventional in air-lubricated thrust bearing can improve significantly the dynamic stiffness of the air film on the bearing significantly [13]. The same goes to oil-lubricated thrust bearing; geometry optimization has been carried out by varying the groove geometrical design by changing the allowable film thickness. This gives better improvement in dynamic stiffness as well [14].

## 2. Numerical Simulation

Modelling is done using Solid Works software while computational fluid dynamic (CFD) calculations are conducted using Ansys Fluent. Simulations were done on two types of models; the first is on a rectangular plane model, and the other is on the surface of a container ship models. For clearer comparisons, simulations are conducted on both models: one with biomimetic shark skin and one without biomimetic shark skin.

### 2.1. Geometry and Meshing

**Figure 2:**
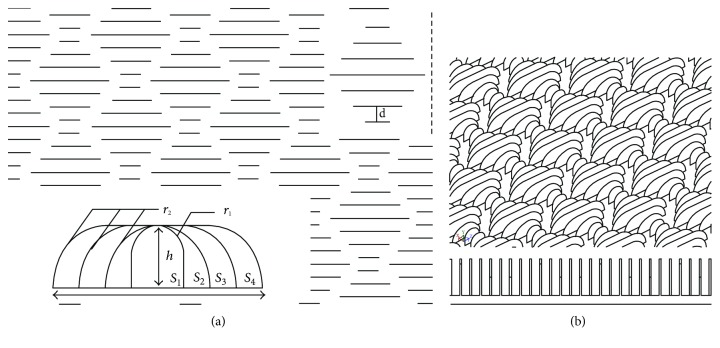
(a) Parameters of riblet structure of biomimetic shark skin. (b) 3D isometric and frontal view of biomimetic shark skin.

Both container ship models have the same boundary conditions as the rectangular plane models. Generic models of planes and container ships are generated by using a 3-dimensional (3D) modelling software. Models used in this study were rectangular plane (RP) with thickness of 1.50 cm and container ship (CS) models, with both models with one without biomimetic riblet structure of shark skin and one with addition of biomimetic riblet shark skin.  Figure 2(a) shows the riblet structure of the shark skin models, and the parameters are as follows: radius, *r*_1_ = 1.0 cm and *r*_2_ = 2.0 cm; riblet height, *h* = 2.0 cm; riblet length, *s*_1_ = 2.0 cm, *s*_2_ = 4.0 cm, *s*_3_ = 6.0 cm, and *s*_4_ = 8.0 cm, respectively; and distance between riblets, *d* = 1.0 cm while Figure 2(b) shows the 3D and frontal view of the proposed riblets. In this study, the RP models are used to study the hydrodynamic effect of the implementation of biomimetic riblet structure of shark skin on surfaces while the CS models are used to study the effect of the biomimetic shark skin to ship. Meshing for all models was generated by using ICEM CFD, and all the computational grids were consisted of fine mesh-unstructured grids of 4 node linear tetrahedrals, 6 node linear wedges, and 5 node linear pyramids. Meshing details for models are as shown in Table 1.

The morphology of a real shark skin is complex 3D geometries and configurations. However, it is still feasible to simulate and study the drag reduction through these complex geometries upon using a powerful supercomputer. Meshing of complex models is a difficult process in CFD. It will consume a lot of time not only to generate those mesh but also to run the simulation itself. These technical assessments of users' designs are still possible for CFD activity; however, these will require massive parallel computing supported by powerful hardware. Numerical solving of complex flows for complex geometries as an attempt of representing the real-like conditions will encompass vast amount of computational power in trying to solve such cases or such fluid's dynamic problems which also reliant on the physical of the model itself. In this case study, it focuses on simplified version shark skin or known as riblet geometry in line to our target of proposing a much more economical and practical modifications for ship hull.

The geometry of the riblets in this paper is proposed through reference of several studies that focus on the study of riblet geometry which is also inspired by shark skin denticle. Riblets are considered as two-dimensional geometry. Real shark skins give complex 3D geometries which lead to cost constrain in fabrication and may not be feasible for mass application. However, this inspired researchers today to focus more on the optimization of this denticle morphology in developing a simpler structure (riblet) as an attempt to achieve the same effect as the real shark skin. There are several different riblet studies which have different riblet structures such as scalloped riblets, sawtooth riblets, blade riblets, L-shaped riblets, U-shaped riblets, and few other riblet geometry [12, 15]. Based on these studies, all of the riblets have the same functionality, however, differ in its percentage of drag-reduction effect due to the structure of the riblet geometry. Different configurations gave different percentage of drag-reduction efficiency. For example, in the review paper written by Dean and Bhusan which discusses on three different riblet designs, namely, scalloped, sawtooth, and blade riblets, which research was done by Bechert et al., it summarizes that blade-type riblets gave the highest maximum value of drag reduction up to 9.9% compared to scalloped and sawtooth riblet type and the value of drag reduction continues to increase parallel to the decrease in the thickness of the riblet. The design proposed for this research was referring to the work of others as a guide in creating a new configuration of the blade-type riblets. The riblet height plays a role in lifting the vortices form while controlling the velocity of the fluid flowing through the surface. In a normal flat plane surface, flowing fluid through the plane will form vortices above the surface that create high shear stresses that lead to drag. Thus, these protruding riblets will help to lift the vortices above the surface and interact only on the tip of the riblets. This allows fluid to flow at lower velocity inside the valley of the riblets, hence, producing lower shear stress. The longitudinal riblets cause velocity fluctuation on the riblet plane to be much lower near the surface compared to the velocity fluctuation on the normal plain surface. This velocity fluctuation continues as fluid flows through the riblet surface since high velocity vortices act on the tip of the riblet; thus, only this localized area will experience high shear stresses [8].

### 2.2. Parameter Set-Up for Flow Simulation

The parameters used are standardized according to the type of models. Inlet velocity for both rectangular plane models is 12.86 m/s which is equivalent to 25 knots, the standard speed of the ship during cruising while other parameters like pressure were set based on the normal room conditions. However, the inlet velocity for container ship models is 128.6 m/s as the models are scaled down to 0.1. Other parameters are summarized as shown in Table 2.

#### 2.2.1. Turbulence Model and Boundary Condition

This research covers an external flow field study on surfaces. To have an acceptable level of simulation accuracy, the selection of turbulence model is very important. In this paper, realizable k-epsilon (k-*ε*) in Ansys Fluent is selected. The turbulence model of k-*ε* consists of two transport equations which signify the properties of turbulence model, and the near-wall treatment with standard wall functions is also implemented in the model. First equation is kinetic term, *k* which functions to determine the turbulent energy production, and the second equation is the turbulent dissipation term, *ε* which functions to determine the scales of the turbulence. Both of these equations signify the turbulent properties of the stream or flow [16]. The k-*ε* turbulence models are adopted to all the models used for this study. In addition to the course of the simulation process, the boundary conditions for all models are treated as follows: (1) A uniform velocity is specified for the flow inlet, where in this study, inlet velocity values, *v* = 12.86 m/s and *v* = 128.6 m/s, are set for RP and CS models, respectively. Inlet velocity values for CS models are calculated based on Reynold's ratio as the scale of the ship model is 0.1 to the scale of real container ship. (2) Pressure value is not specified at the outflow boundary condition as the pressure outlet is calculated depending on the interactions of the ship hull surface and fluid interior of the computational flow field. (3) Other than the inlet and outlet boundary conditions, the surfaces at the edge of the computational field are treated as walls with no slip condition. As for container ship models, addition of inflation layers is applied to the hull of both ship models. In this study, the biomimetic shark skin is applied on frontal and rear end of the ship and both ship models are configured with symmetrical boundary condition. Thus, only half of the ship model is simulated as a single phase with k-*ε* turbulence flow and the computational domain only cover on the bottom part of the ship where the biomimetic riblet structure shark skins are applied.

## 3. Results and Discussion

The analysis of the results for every section is based on the flow trajectories or contour lines on the particular models. For all the models used in this research, the value of average, minimum, and maximum of any parameters used is calculated or determined by using calculator function which is provided in Ansys.

### 3.1. Flow Field Analysis on RP Models

#### 3.1.1. Analysis of Velocity Field

**Figure 3:**
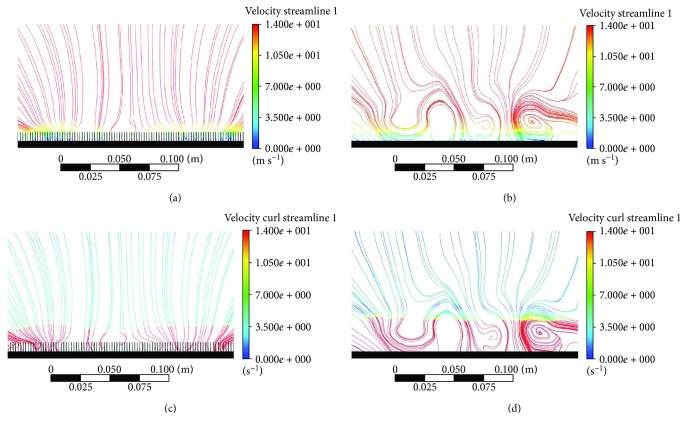
(a) Cross-section of velocity streamline of riblet RP. (b) Cross-section of velocity streamline of plain RP. (c) Cross-section of velocity curl of riblet RP. (d) Cross-section of velocity curl of plain RP.

Figure 3 shows the velocity profile in cross-section of the flow field over the plain and riblet RP models. The velocity varies in different parts of the flow field. Figures 3(a) and 3(b) show the vertical cross-section of velocity streamline on the riblets and plain of RP models, respectively. Based on the result, the average velocity for riblet RP is 10.79 m/s with values of the maximum up to 14.41 m/s and the minimum of 0.73 m/s. Meanwhile for plain RP, the average velocity is 13.06 m/s while the maximum velocity and minimum velocity are approximately 13.85 m/s and 9.07 m/s, respectively. The vertical cross-section of both surface planes gave different form of vorticity. Figure 3 shows that vortices are present in both surfaces, however differ in size and position due to riblet geometry. As mentioned by Pu et al., riblets help in translating the streamwise vortices that will reduce the vortex discharge and turbulence experienced at the outer layer [17].

**Figure 4:**
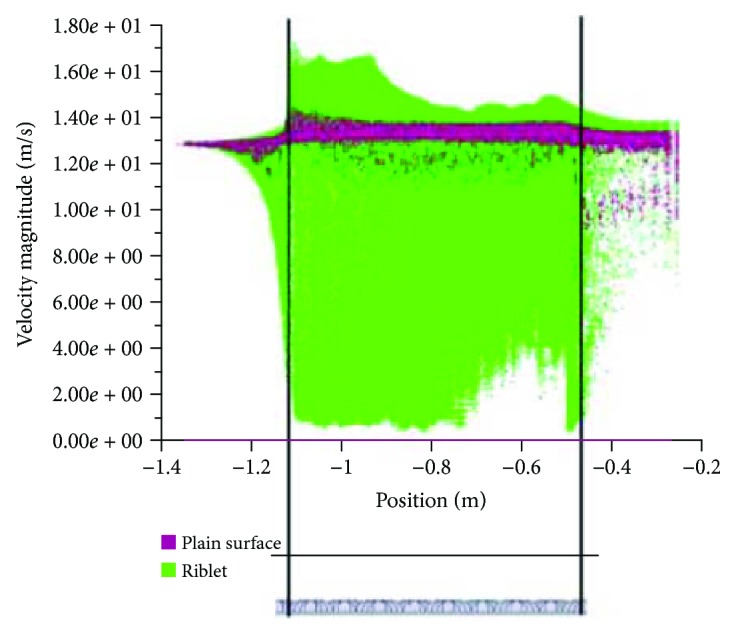
XY plot of velocity magnitude (m/s) to the direction vector (m) of both RP models.

From the results obtained, larger vortices formed above the plain RP model vicinity are shown in Figure 3(d) indicating that high velocity flow is acting above the surface. However, in Figure 3(c), the vortices are lifted off the surface due to longitudinal riblet geometry causing fluctuation in velocity inside riblet valley which leads to an even lower velocity near the surface than those velocities on plain surface and only small vortices can be seen acting on the tip of the riblet RP model [18]. Fluctuation in velocity can be seen obviously in Figure 4 where the profile of *x*-axis velocity magnitude to the direction vector of the flow on both surfaces was extracted and plotted.

#### 3.1.2. Analysis of Wall Shear Stress

**Figure 5:**
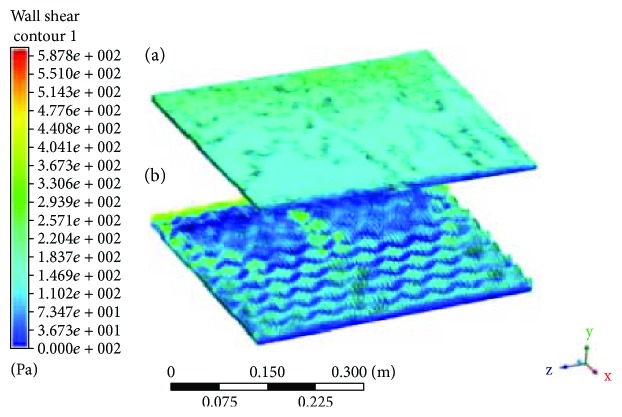
(a) Contour of wall shear stress on plain RP. (b) Contour of wall shear stress on biomimetic riblet RP.

The changes in the velocity distribution along the flow field lead to a reduction in wall shear stress. As velocity flow profile inside the valley of the riblet RP model is lower, it indirectly reduces the shear stresses experienced by the model. Since higher vortices are only experienced on the tip of the riblets, these cause only small amount of high shear stress interacted with the plane as shown in Figure 5. Reduction in wall shear stress can lead to the reduction of drag experienced by a moving vehicle or animal as every moving object experienced drag force. Drag reduces as wall shear stress reduces or for some cases, by separation control as mention by Bechert et al. [12, 18]. Wall shear stress on plain RP is nearly uniformed with average wall shear stress of 129.9 Pa. However, for riblet RP, the average wall shear stress takes approximately 112.8 Pa with the same flow direction, with the maximum of 880.8 Pa on the tip of the riblets comparatively larger than the plain surface; however, on the valley, wall shear stress is so much lower compared to the plain RP. Thus, this shows a reduction in drag forces as riblet RP gave lower wall shear stresses compared to the plain RP.

### 3.2. Flow Field Analysis on CS Models

#### 3.2.1. Analysis of Wall Shear Stress

**Figure 6:**
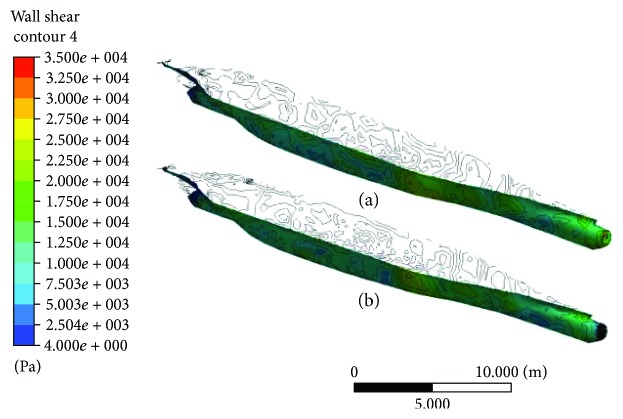
(a) Contour of wall shear stress on container ship model without biomimetic shark skin. (b) Contour of wall shear stress on container ship model with biomimetic shark skin.

**Figure 7:**
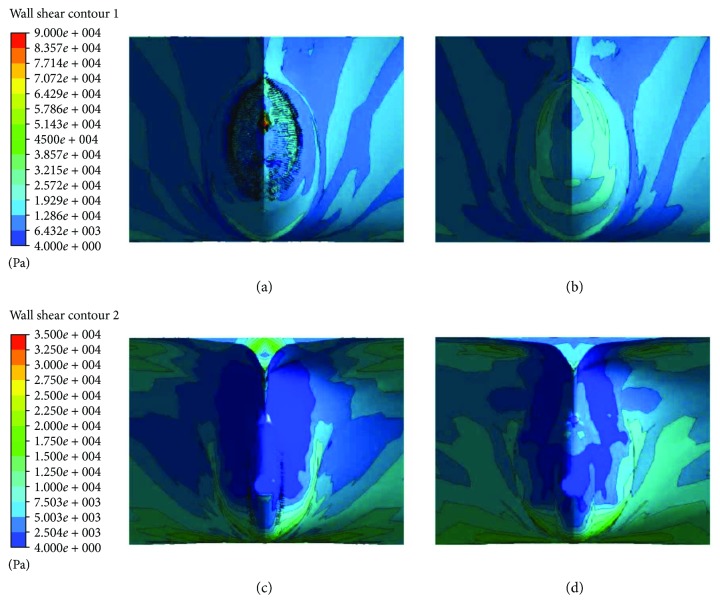
(a) Contour of wall shear stress on frontal ship model with biomimetic shark skin. (b) Contour of wall shear stress on frontal plain ship model. (c) Contour of wall shear stress on rear ship model with biomimetic shark skin. (d) Contour of wall shear stress on rear plain ship model.

Figure 6 shows the contour of the wall shear stresses on both CS models with biomimetic shark skin and without shark skin. Both ships are in the same direction of flow. Contour of wall shear stress on both bodies of the ships is nearly the same and uniform. To further evaluate the shear stresses, the results are focused on the frontal and rear end of the ships on plain vicinity and on vicinity where biomimetic shark skin is applied as shown in Figure 7. Based on the results obtained, it clearly shows that there are reductions in wall shear stresses in both areas: frontal and rear end. The implementation of the biomimetic shark skin on the particular area gives twice better result compared to the plain ship model. There is a slight increase of shear stresses on the tip of the bow as shown in Figure 7(a). However, it reduces over the biomimetic shark skin and even decreases lower than the frontal CS model without shark skin. Figures 7(c) and 7(d) show the contour results of the rear end for CS models with biomimetic and without biomimetic shark skin, respectively. The wall shear stress for the rear end with biomimetic shark skin is lower and spreads uniformly at the average of 4 Pa to 2500 Pa compared to plain rear end CS model with range between 4 Pa and 7500 Pa. As mention earlier in the analysis of wall shear stress for RP models, reduction in wall shear stress can lead to the reduction of drag force [18].

#### 3.2.2. Analysis of Turbulent Kinetic Energy

**Figure 8:**
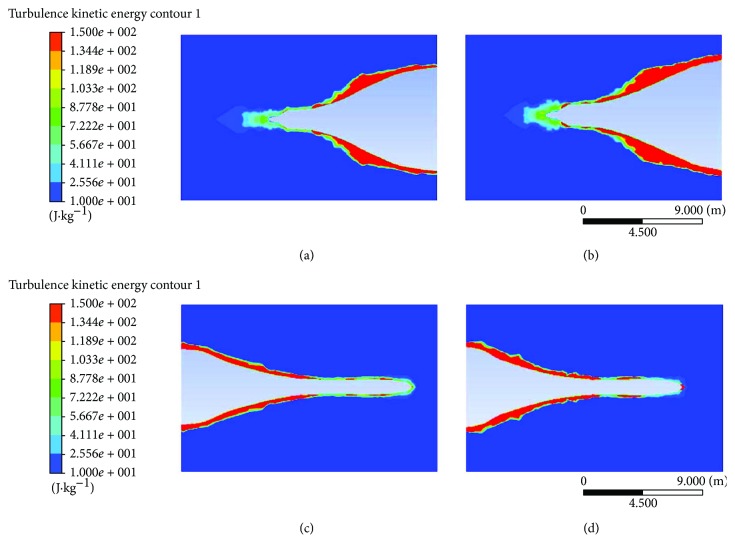
(a) TKE on rear end model of container ship with biomimetic shark skin. (b) TKE on rear end model of plain ship. (c) TKE on front model of container ship with biomimetic shark skin. (d) TKE on front model of plain ship.

Energy content of eddies in turbulent flow is known as turbulent kinetic energy (TKE). Energy content of eddies is proportional to the size of it. The greater the size, the more energy content of eddies. The high turbulent energy extraction from the mean flow leads to high region of turbulent kinetic energy [19]. The result analyses are focused on the area which applied with biomimetic shark skin as shown in Figure 8. The kinetic energy size for rear end CS model with biomimetic shark skin extends slightly longer and thinner compared to plain rear end CS model. There are also reductions in turbulent kinetic energy region as TKE on both frontal and rear end of ship model with biomimetic shark skin shows lower than that on the plain model. Decrease of TKE region indicates the reduction of energy content eddies acting on the ship, improving the fluid flow around the ship, making it easier for the ship to glide through the water. It can be observed that the presence of the biomimetic shark skin can help in reducing the size of kinetic energy region on both areas for the CS model with biomimetic shark skin.

**Figure 9:**
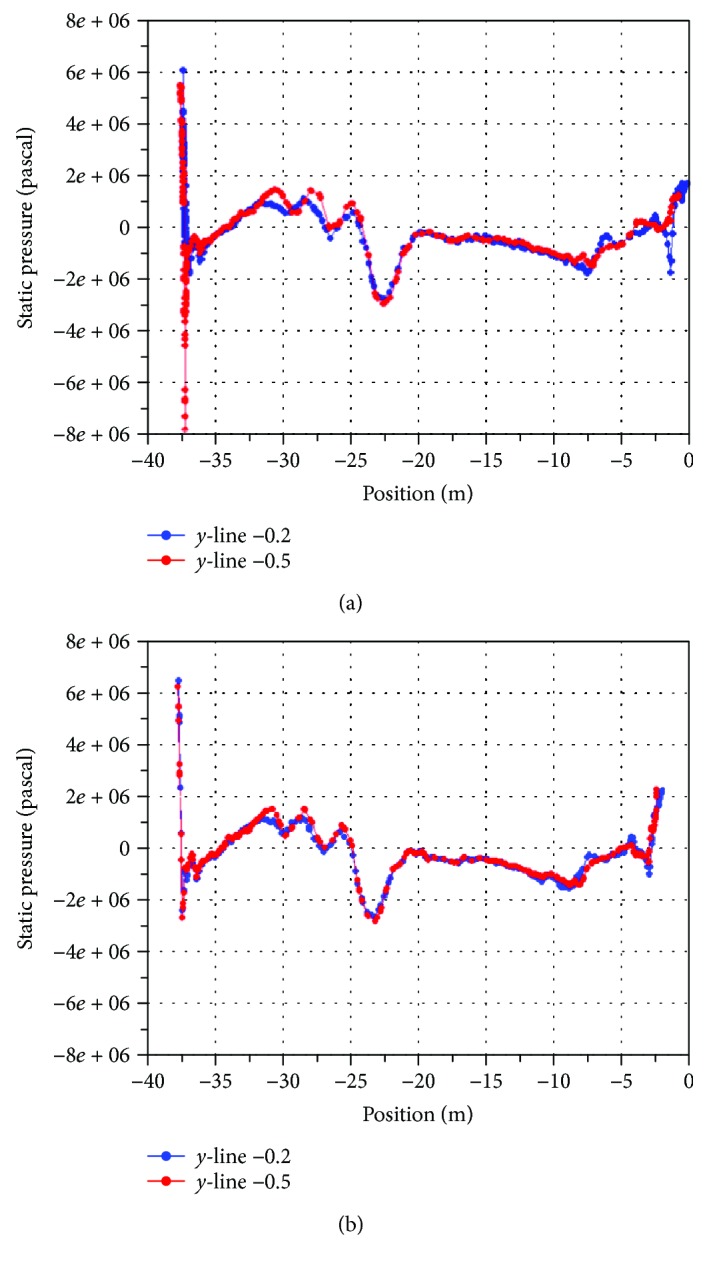
(a) Pressure, isoline on CS model with biomimetic shark skin. (b) Pressure, isoline on CS model without biomimetic shark skin.

Figure 9 shows the pressure graph of both CS models, with and without biomimetic shark skin on two different isoline of *y*-axis = 0.2 and *y*-axis = 0.5 about the container ships. These lines are constructed to further analyze on pressure acting on the model with relation to force and wall shear stress acting on it. Based on the graphs shown in Figure 9, ranges of all isolines for pressure acting on the CS model without BSS and with BSS are between positions of −30 m to 0 m. Comparing both graphs, the CS model with biomimetic shark skin gave lower value of both the maximum and minimum pressure compared to CS model without biomimetic shark skin. The reduction in pressure can be seen in both implementation areas, frontal vicinity and rear vicinity of the CS models. However, pressure acting on the other parts of the body on both models is almost similar and uniform. This pressure form is dependent on the amount of force exerted to it. High force exerted contributes high amount of pressure, and this can be related through the definition of the pressure itself.

**Figure 10:**
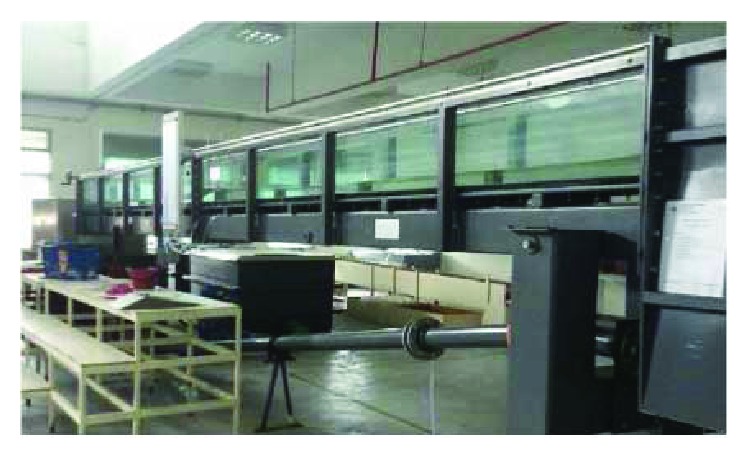
8-meter flow channel, Hydrology Lab of UNIMAS.

The results in Table 3 show the percentage of improvement of drag reduction for CS models with biomimetic shark skin to a normal plain model ship. The authors are now actively setting up an experimental procedure to validate the numerical results obtain in this study which the experimental set up will be carried out using a water flow channel as shown in Figure 10 conducted in the Hydrology Lab of Universiti Malaysia Sarawak (UNIMAS). The results of drag reduction in this study is summarized as below.

## 4. Conclusion

Design modification with biomimetic shark skin has numerically proven that the biomimetic shark skin could give improvement to both models: rectangular plane and container ship models in terms of reduction in wall shear stress, reduction in drag force, and improved fluid flow around the ship. The coefficient of drag reduced by 3.75% and gave the reduction of 3.89% in drag force experienced by container ship model. Since the numerical study gave some encouraging results, we can further improve this research by conducting experimental procedure for validation. This modification might give better impact to shipping or marine industries around the world.

## Figures and Tables

**Table 1:** Meshing details for each models.

Models	Number of nodes	Number of elements	Maximum element quality
RP with biomimetic shark skin	275975	1447984	0.99980
RP without biomimetic shark skin	16927	84922	0.99984
CS with biomimetic shark skin	171265	549536	0.99978
CS without biomimetic shark skin	53862	224310	0.99980

**Table 2:** Parameters set-up for flow simulation.

Models	Plain models	Biomimetic shark skin
Pressure (Pa)	101325	
Viscosity (kg/m·s)	1.09 × 10^−3^	
Density (kg/m^3^)	1025	
Gravitational acceleration (m/s^2^)	−9.81	
Type of fluid	Seawater	
Type of flow	Turbulence	

**Table 3:** Improvement percentage.

Container ship models	With biomimetic shark skin, *a*	Without biomimetic shark skin, *b*	Difference, *c* = *a* – *b*	Percentage (*c/a*) × 100%
Drag coefficient, *C*_d_	0.077	0.080	0.003	3.75
Drag force (N)	29434	30581	1147	3.89
